# Identification of a proliferator-activated receptor-γ antagonist for the treatment of type 2 diabetes mellitus

**DOI:** 10.3892/etm.2014.2096

**Published:** 2014-12-01

**Authors:** REN WANG, LIHUA DAI, JINJIN CHEN

**Affiliations:** 1 Department of Ultrasonography, Shanghai Sixth People’s Hospital, Affiliated to Shanghai Jiao Tong University, Shanghai 200233, P.R. China; 2 Department of Emergency, Xinhua Hospital, Affiliated to Shanghai Jiao Tong University School of Medicine, Shanghai 200092, P.R. China; 3 Department of Child Health Care, Shanghai Children’s Hospital Affiliated to Shanghai Jiao Tong University, Shanghai 200040, P.R. China

**Keywords:** type 2 diabetes, peroxisome proliferator-activated receptor-γ antagonist, lipid metabolism-related genes

## Abstract

In the present study, a novel antagonist of the peroxisome proliferator-activated receptor-γ (PPARγ) was screened and identified, and a cell-based evaluation of the biological activity of this PPARγ antagonist was conducted. The aim of the study was to produce results that may provide a foundation for the development of a novel compound in the treatment of type 2 diabetes mellitus. Since obesity is the main cause of insulin resistance and type 2 diabetes, identifying a new reagent that is able to inhibit adipocyte differentiation and lipid accumulation is a feasible method of developing novel anti-diabetes drugs. The PPARγ antagonist was screened using a mammalian one-hybrid system and transcriptional activation. The effects of the compound on adipocyte differentiation were investigated by staining the preadipocytes with Oil Red O. In addition, the effects of the compound on the expression levels of genes associated with lipid metabolism were detected using quantitative polymerase chain reaction on differentiated mature 3T3-L1 adipocytes. As a PPARγ antagonist, N-((1H-benzo[d]imidazol-2-yl)methyl) aniline (Compound Q) was shown to depress the transcriptional activity and coactivator recruitment of PPARγ, as well as preadipocyte differentiation, in a concentration-dependent manner. The compound was also shown to decrease the expression levels of genes associated with PPARγ-regulated lipid metabolism. In conclusion, the compound screening platform was demonstrated to be valid, and the present study identified a novel PPARγ antagonist that was shown to effectively reduce the rate of adipocyte differentiation and the expression of genes associated with lipid metabolism.

## Introduction

Type 2 diabetes mellitus is a metabolic syndrome with a complex pathogenesis, which is currently exhibiting an increasing morbidity rate each year. However, there is no effective therapeutic drug (1). Type 2 diabetes mellitus is predominantly caused by abnormal sugar and lipid metabolism, due to the relative deficiency of insulin (insufficient insulin secretion and insulin resistance), or excessive glucagon levels. Clinically, patients present with hyperglycemia and hyperlipemia (2). While having a large impact on patient quality of life, type 2 diabetes mellitus also becomes a burden for the development of a modern economic society. The American Diabetes Association and the European Association for the Study of Diabetes proposed that the identification of a glycosylated hemoglobin level of >7% among patients may be used as an indication of undesired blood glucose control (3). The existing oral antidiabetics demonstrate severe side-effects, and treatment with insulin increases the risk of weight gain and hypoglycemia. Therefore, the development of novel and effective methods for the treatment of type 2 diabetes mellitus, or the identification of novel small molecular drugs, is urgently required (4). Peroxisome proliferator-activated receptor-γ (PPARγ) is a member of the nuclear receptor super family, which plays an important role in regulating glucolipid metabolism (5). PPARγ is a ligand-dependent nuclear receptor (6). Following the integration of ligand binding and activation, the receptor forms a heterodimer with the retinoid X receptor-α (RXRα), and regulates the expression levels of associated genes on the peroxisome proliferator response element (PPRE) (7).

Among the clinical drugs used in the treatment of type 2 diabetes mellitus, thiazolidinediones, such as rosiglitazone (Ros) and pioglitazone, can be used as PPARγ agonists to significantly reduce hypoglycemia and improve the sensitivity to the insulin pathway. However, these drugs have strong side-effects concurrently, including weight gain or increased risks of cardiovascular diseases, which constrain their use in clinical practice (8). However, in recent years, studies have demonstrated that a PPARγ gene knockout or intermediate depression of PPARγ activity (9) can relieve the insulin resistance induced by a high fat diet (10). This treatment method also avoids the side-effects that are observed with a PPARγ full agonist (11). Therefore, the screening of small molecular compounds based on PPARγ antagonists is a key strategy for the identification of novel compounds for the treatment of type 2 diabetes mellitus (12). Currently, there are a limited number of studies investigating PPARγ antagonists. Therefore, the development of novel drugs for the treatment of type 2 diabetes mellitus based on PPARγ antagonists is of great importance (13).

In the present study, transcriptional activation and a mammalian one-hybrid system were applied to screen for the PPARγ antagonist N-((1H-benzo[d]imidazol-2-yl)methyl) aniline, referred to as ‘Compound Q’. Furthermore, the effects of this antagonist on the expression levels of PPARγ-regulated lipid metabolism genes and the differentiation of adipocytes were studied in order to develop novel reagents with the potential to ameliorate obesity and type 2 diabetes.

## Materials and methods

### Cell culture of the 293T and 3T3-L1 preadipocyte cell lines

A 293T cell line and 3T3-L1 preadipocyte cell line (American Type Culture Collection, Manassas, VA, USA) were cultured in Dulbecco’s modified Eagle’s medium (DMEM; Gibco Life Technologies, Grand Island, NY, USA), containing 10% fetal bovine serum (Gibco Life Technologies) and 1% penicillin-streptomycin, at 37°C in saturated humidity with 5% CO_2_. The culture solution of the cells was exchanged on a daily basis and trypsinization was processed every two days. Cells at a logarithmic growth phase were seeded for the experiment.

### Luciferase activity test

Effects of Compound Q (J&K Chemical Co., Ltd., Shanghai, China) on the coactivator recruitment of PPARγ and the transcriptional activity of the RXRα-PPARγ heterodimer were analyzed. At a logarithmic phase of cell growth, the 293T cells were seeded in a 24-well cell culture plate. When the cells reached a density of 50–70%, the medium was replaced with serum-free DMEM. Plasmids were transfected into the cells using a Calcium Phosphate Transfection kit (Nanjing Jiancheng Bioengineering Institute, Nanjing, China). To assess the ability of the compound for PPARγ coactivator recruitment, PPARγ-LBD, UAS-TK-Luc reporter and an internal reference plasmid, pRL-SV40 (all from Promega Corporation, Madison, WI, USA), were transfected in the 293T cells. When assaying the effects of the compound on the transcriptional activity of the RXRα-PPARγ heterodimer, the full-length plasmids of RXRα and PPARγ, PPRE-Luc and the internal reference plasmid, pRL-SV40, were transfected into the 293T cells. After 6 h of transfection, the medium was replaced with complete medium containing 10% fetal bovine serum. In addition, Compound Q was added to the medium for 18 h of culturing. The medium from the culture dish was removed and the plates were washed with phosphate-buffered saline (PBS). To each well, 100 μl cell lysis buffer was added and incubated at 37°C for 20 min to enable cell lysis. Next, with reference to the instructions in the luciferase kit (Dual-Luciferase^®^ Reporter Assay system; Promega Corporation), the activity of the firefly luciferase and internal reference, fluorescein, was analyzed.

### 3T3-L1 adipocyte differentiation experiment

A 3T3-L1 preadipocyte differentiation assay was conducted according to the reported classic procedure (14). Briefly, 3T3-L1 preadipocytes at a logarithmic growth phase were seeded into six-well cell culture plates. The cell density was allowed to reach 100%, and this time point was set as day 0. On day 4, the complete medium containing 0.115 mg/l methylisobutylxanthine (MIX), 0.39 mg/l dexamethasone (DEX) and 1 mg/l insulin was cultured for three days. The solution was further stimulated through the removal of old medium and the addition of fresh medium with 1 mg/l insulin, which was cultured for an additional three days. Following these stimulations, the differentiated adipocytes were cultured in normal medium (DMEM containing 10% FBS) for 1–2 days prior to carrying out further experiments.

To observe the effects of Compound Q on adipocyte differentiation, a negative control [dimethyl sulfoxide (DMSO)], positive control (Ros) and Compound Q at various concentrations (1, 10 and 20 μM) were added to the differentiation medium. After adipocyte stimulation for 6 days, the differentiation medium was removed and the cells were washed three times with PBS. The solution was stained using a Oil Red O kit (Nanjing Jiancheng Bioengineering Institute) according to manufacturer’s protocol, to investigate the lipid accumulation in adipocytes. Images were photographed for observation using a BX 50 microscope (Olympus Corporation, Tokyo, Japan) (15).

### Quantitative polymerase chain reaction (PCR)

Mature 3T3-L1 adipocytes that had undergone differentiation were cultured in six-well plates and processed with different compounds, including a negative control (DMSO), positive control (Ros) and Compound Q at various concentrations (1, 10 and 20 μM), that were added to the complete medium for 24 h. Total RNA was extracted using TRIzol reagent (Invitrogen Life Technologies, Carlsbad, CA, USA), and reversed transcribed to cDNA using a PrimeScript^TM^ RT kit (Takara Biotechnology, Co., Ltd., Dalian, China). SYBR Green Real Time PCR Master Mix (Toyobo Co., Ltd., Tokyo, Japan) was used for PCR, which was performed in a DNA Engine Opticon 2 device (Bio-Rad Laboratories, Inc., Hercules, CA, USA). The primers used in the experiment were as follows: GAPDH forward, 5′-GTATGACTCCACTCACGGCAAA-3′ and reverse, 5′-GGTCTCGCTCCTGGAAGATG-3′; fatty acid synthase (FAS) forward, 5′-CTGAGATCCCAGCACTTCTTGA-3′ and reverse, 5′-GCCTCCGAAGCCAAATGAG-3′; CCAAT/enhancer-binding protein-α (C/EBPα) forward, 5′-CTGAGATCCCAGCACTTCTTGA-3′ and reverse, 5′-CACGGCTCAGCTGTTCCA-3′; adipocyte fatty acid binding protein 2 (aP2) forward, 5′-CATGGCCAAGCC CAACAT-3′ and reverse, 5′-CGCCCAGTTTGAAGG AAATC-3′; HMG-CoA forward, 5′-CATGCAGATTCT GGCAGTCAGT-3′ and reverse, 5′-CGGCTTCACAAACCA CAGTCT-3′. These genes were selected as they are PPARγ-regulated and the main genes involved in lipid metabolism. All the samples were assayed according to the manufacturer’s instructions.

### Transcriptional activation and mammalian one-hybrid system

A mammalian one-hybrid system was carried out to screen compounds which were able to directly bind and influence the recruitment of co-activators to PPARγ-LBD. In the mammalian one-hybrid system, the Gal4/UAS system was used to investigate the activity regulation of PPARγ, whether the co-transfection of Gal4-PPARγ-LBD and UAS-TK-Luc into 293T cells was able to express the Gal4-PPARγ-LBD protein, and whether the potential compound was able to activate or inhibit PPARγ-LBD and its ability to influence the expression of TK-Luc. A transcriptional activation system was also carried out to investigate the transcription activity of PPARγ. As the co-transfection of pGL3-PPRE-Luc and pcDNA3.1-PPARγ into 293T cells results in PPARγ expression, whether the potential compound was able to activate or inhibit PPARγ activity, and increase or decrease the transcription of PPRE-Luc was investigated. In the mammalian one-hybrid and transcriptional activation systems, SV40 was used as the control for transfection efficiency.

### Statistical analysis

Data are shown as mean ± standard error of the mean. A student’s t-test was performed for the comparison of two groups and one-way analysis of variance was carried out for the comparison of >2 groups by GraphPad Prism 5 software (GraphPad, San Diego, CA, USA). P<0.05 was considered to indicate a statistically significant difference.

## Results

### Compound Q as a PPARγ antagonist

**Figure 1:**
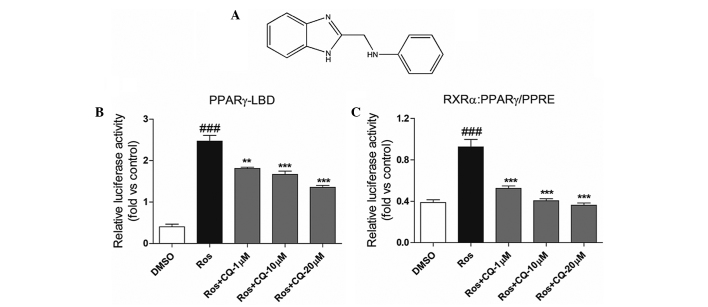
(A) Structural formula of Compound Q. Compound Q was shown to depress the activity of Ros-activated (B) PPARγ-LBD-Luc and (C) RXRα:PPARγ-PPRE-Luc in a concentration-dependent manner. Ros, a known PPARγ agonist, is able to activate PPARγ-LBD and increase the transcription of PPRE-Luc. As compared with Ros treatment, the combination of Compund Q and Ros decreased Ros-induced luciferase activity in the mammal one-hybrid and transcriptional activation systems, suggesting that Compund Q is a PPARγ antagonist and can inhibit the transcriptional activity of PPARγ. ^###^P<0.001 compared to DMSO group; ^**^P<0.01 and ^***^P<0.001, respectively, compared to Ros group. Ros, rosiglitazone; DMSO, dimethyl sulfoxide; PPARγ, peroxisome proliferator-activated receptor-γ; Luc, luciferase; PPRE, peroxisome proliferator response element; RXRα, retinoid X receptor-α; CQ, Compound Q.

As shown in Fig. 1, a mammalian one-hybrid method using a luciferase-reporter system was used for the experiment. Compound Q (Fig. 1A) was shown to depress the coactivator recruitment of PPARγ in a concentration-dependent manner (Fig 1B). In addition, with regard to transcriptional activation, Compound Q was revealed to reverse the transcriptional activity of the Ros-activated RXRα-PPARγ heterodimer in a concentration-dependent manner (Fig. 1C). The observations indicated that as a PPARγ antagonist, Compound Q is able to reduced the transcriptional activity of the RXRα-PPARγ heterodimer.

### Compound Q depresses 3T3-L1 preadipocyte differentiation in a concentration-dependent manner

As PPARγ is a key moderator of lipid metabolism and a vital gene involved in adipocyte differentiation, a previous study reported that PPARγ agonists, such as Ros, are able to significantly promote adipocyte differentiation (14). Thus, the present study further investigated the effects of Compound Q on adipocyte differentiation.

**Figure 2:**
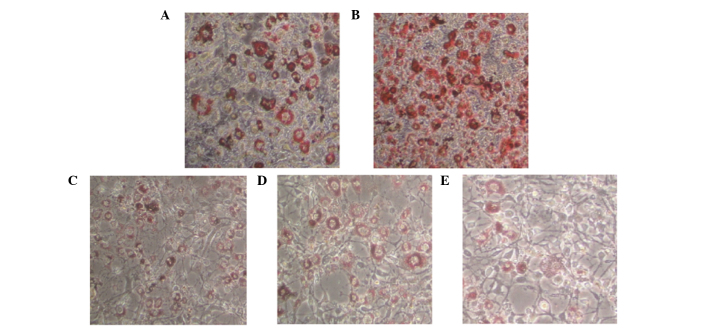
Compound Q was shown to reduce the rate of adipocyte differentiation in a concentration-dependent manner. (A) DMSO; (B) Ros; (C) Compound Q (1 μM); (D) Compound Q (10 μM); and (E) Compound Q (20 μM). DMSO was used as a negative control, while Ros was used as a positive control, since the drug is known to significantly enhance adipocyte differentiation. Ros, rosiglitazone; DMSO, dimethyl sulfoxide. Magnification, ×200.

Compound Q at various concentrations, or the negative or positive control, were added to the differentiation medium. At the end of the differentiation assay, staining with Oil Red O demonstrated that the positive control, Ros, was able to significantly enhance adipocyte differentiation, while Compound Q decreased the formation of grease drops in adipocytes and the formation of adipocytes in a concentration-dependent manner (Fig. 2). This indicates that Compound Q has the potential to inhibit triglyceride accumulation in adipose tissue and ameliorate obesity in obese patients.

### Compound Q decreases the expression levels of genes associated with lipid metabolism

**Figure 3:**
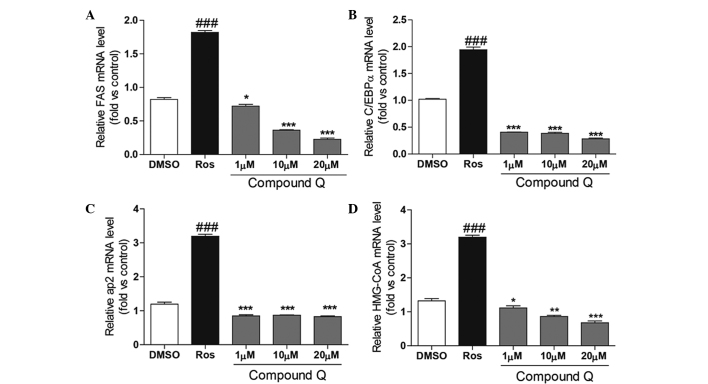
Differentiated mature adipocytes were cultured for 24 h in a medium containing Compound Q at various concentrations, a positive control (Ros) or a negative control (DMSO). Compound Q was shown to decrease the expression levels of (A) FAS, (B) C/EBPα, (C) aP2 and (D) HMG-CoA in a concentration-dependent manner, while Ros was found to increase the expression levels. ^###^P<0.001, ^*^P<0.05, ^**^P<0.01 and ^***^P<0.001. Ros, rosiglitazone; DMSO, dimethyl sulfoxide; FAS, fatty acid synthase; C/EBPα, CCAAT/enhancer-binding protein-α; aP2, adipocyte fatty acid binding protein 2.

Adipocyte differentiation is a complex process involving the regulation of multiple genes. In order to study the mechanism underlying the Compound Q-induced depression of adipocyte differentiation, quantitative PCR was used to analyze the effects of Compound Q on the expression levels of key genes involved in lipid metabolism. As shown in Fig. 3, following the culture of adipocytes for 24 h with Compound Q at various concentrations, the positive control (Ros) or the negative control (DMSO), the total RNA of the different cells was extracted. Following quantitative PCR, Ros was shown to enhance the expression levels of FAS, C/EBPα, aP2 and HMG-CoA. By contrast, treatment with Compound Q decreased the expression levels of the genes associated with lipid metabolism in a concentration-dependent manner.

## Discussion

Type 2 diabetes mellitus has become a common disease with a high morbidity rate in modern society. Patients mainly present with hyperglycemia and hyperlipemia, as well as a range of complications induced by hyperglycemia in the later stages, such as diabetic nephropathy and diabetic foot (16). Hyperglycemia is primarily caused by the failure of pancreatic β cells to sufficiently secrete insulin and compensate for the insulin resistance of tissues. This results in reduced glucose absorption and increased lipidolysis (17). Hyperglycemia may also be the result of excessive glucagon secretion by pancreatic α cells, which leads to an increase in gluconeogenesis. The condition ultimately results in abnormal glucolipid metabolism of the organism (18). Clinically, oral antidiabetics have diverse side-effects, and treatment with insulin has revealed risks of weight gain and hypoglycemia (19,20).

Currently, oral antidiabetics, including Ros and pioglitazone of the thiazolidinedione class of medications, perform hypoglycemic effects and increase insulin sensitivity by activating the nuclear receptor, PPARγ (21). However, these drugs have severe side-effects, including weight gain and increased risks of cardiovascular diseases (22). In recent years, research has found that decreasing the activity of PPARγ by constructing PPARγ gene knockout models or inducing mutations at PPARγ activity sites can relieve the insulin resistance induced by a high fat diet (23).

Based on the aforementioned observations, the present study adopted a mammalian one-hybrid method with transcriptional activation to identify that Compound Q, as a PPARγ antagonist, is able to reduce PPARγ coactivator recruitment and reverse Ros-activated RXRα-PPARγ transcriptional activity in a concentration-dependent manner. In the cell activity assay, Compound Q was found to reduce the formation of grease drops in adipocytes and the formation of adipocytes in a concentration-dependent manner. With further investigation into the underlying mechanism, Compound Q was revealed to decrease the rate of differentiation by decreasing the expression levels of key genes involved in lipid metabolism. Therefore, the effects of Compound Q in depressing adipocyte differentiation and regulating the expression levels of genes associated with lipid metabolism indicate the potential of the compound for weight loss and lipid metabolism regulation therapy.

In conclusion, the present study investigated a PPARγ antagonist, and the results provide a new understanding and research basis for the future investigation into novel small molecular compounds with fewer side-effects for the treatment of type 2 diabetes mellitus. Compound Q is a small molecular compound that was demonstrated to be a significant active compound. However, the biological activity of Compound Q *in vivo* requires further evaluation.
